# Systemwide Evaluation of Field Medicine Programs in Los Angeles County: Protocol for a Mixed Methods Longitudinal Observational Study

**DOI:** 10.2196/94248

**Published:** 2026-10-14

**Authors:** Randall Kuhn, Jessie Chien, Irene Del Mastro N, Sonali Saluja, Amanda Landrian Gonzalez, Geoffrey Cui-Naudet, Gisele Corletto, Sara Ozuna, Brian P Zunner-Keating, Catherine M Weaver, Coley King, Charles Robinson, Benjamin F Henwood

**Affiliations:** 1 Department of Community Health Sciences, Fielding School of Public Health, University of California, Los Angeles, 650 Charles E. Young Dr. South, 16-035 Center for Health Sciences, Los Angeles, CA, 90095, United States, 1 303 928 9111; 2 California Center for Population Research, University of California, Los Angeles, Los Angeles, CA, United States; 3 Department of Sociology, University of California, Los Angeles, Los Angeles, CA, United States; 4 Healthcare in Action, Long Beach, CA, United States; 5 USC Suzanne Dworak-Peck School of Social Work, University of Southern California, Los Angeles, CA, United States; 6 Homeless Healthcare Collaborative, UCLA Health, University of California, Los Angeles, Los Angeles, CA, United States; 7 Homeless Services, Venice Family Clinic, Los Angeles, CA, United States; 8 LA Care Health Plan, Los Angeles, CA, United States

**Keywords:** homelessness, field medicine, longitudinal research, mixed methods, street medicine

## Abstract

**Background:**

People experiencing homelessness face high morbidity and unmet health care needs. To address these gaps, health care providers across the United States are increasingly adopting “field medicine” models that deliver mobile health services in shelters, homeless encampments, mobile clinics, and other community settings. Despite their expanding use, systematic evaluations of field medicine programs remain limited.

**Objective:**

This paper aims to describe a protocol for a mixed methods evaluation of field medicine for people experiencing homelessness across Los Angeles (LA) County, California.

**Methods:**

People experiencing homelessness receiving field medicine were recruited into an ongoing longitudinal study of LA County’s unhoused population; a subset of participants in the existing probability-sampled cohort serves as the comparison group. Using monthly online survey data and a quasi-experimental design, we examine who accesses field medicine, how patients use and perceive care, and the impact of field medicine on health and service engagement. We also conduct participant observation of field medicine teams to document patient-provider interactions, care coordination, and carry out semistructured interviews with providers, patients, and nonpatients. Quantitative survey and qualitative findings will be integrated to identify convergence, complementarity, and explanatory insights.

**Results:**

Recruitment of people experiencing homelessness receiving field medicine occurred between August 2024 and October 2025. Among 847 individuals referred from field medicine, 749 (88.4%) were eligible, and 436 (51.5%) completed the first monthly survey and were enrolled. For the comparison group, 902 (63.8%) of the 1413 participants ever enrolled in the existing cohort completed a survey during the field medicine recruitment period and met eligibility criteria. Participant observation included 82 field visits (≈500 h) across diverse service locations and more than 300 patient-clinician interactions. Semistructured interviews were conducted with 15 (30%) providers, 23 (46%) field medicine patients, and 12 (24%) nonpatients.

**Conclusions:**

This study represents one of the first large-scale mixed methods evaluations of field medicine. With increasing health threats from criminalization, climate-related events, and other socioenvironmental hazards, field medicine may mitigate health risks and improve systems engagement for people experiencing homelessness. Findings will provide rigorous evidence to inform service delivery and policy decisions.

## Introduction

### Background

Homelessness is associated with a loss of up to 20 years of life expectancy, resulting from a combination of adverse exposures and inadequate health care [1-3]. Unhoused people who are unsheltered—meaning they sleep outdoors, in vehicles, tents, or other areas not fit for human habitation—have even poorer health outcomes [4]. In California, home to 25% of the total US unsheltered homeless population, health care providers have created bold new initiatives to provide broad mobile health care services to people experiencing homelessness through a “field medicine” approach. Field-based medicine (hereinafter referred to as “field medicine”) builds on existing “street medicine” approaches, in which health care providers meet patients where they are, both physically and in terms of desired level of engagement, but incorporates a wider range of intervention sites, including shelters, mobile clinics, and pop-up clinics [5,6].

There have been few systematic evaluations of street medicine programs [7,8]. A main challenge to evaluating street medicine is that programs often provide a wide range of curative and preventative services that cannot easily be summarized or categorized as being part of a single “street medicine” intervention [9-11]. An emerging set of studies has evaluated the delivery of specific services, such as hypertension treatment and medication for opioid use disorder, with promising results, but these studies only indirectly address the effectiveness of the model itself [12-14]. More recently, evaluations of comprehensive street medicine programs, including postdischarge transitional care models, have reported encouraging findings while emphasizing the need for more rigorous study designs and longer-term outcome evaluation [15]. Furthermore, street medicine has been variably defined to sometimes include mobile clinics, which require people experiencing homelessness to travel to a fixed or rotating service location, as opposed to teams that deliver care directly to individuals in the places where they are living. In 2023, the Centers for Medicare & Medicaid Services made street medicine a billable service, providing clearer guidance on what qualifies as street medicine. However, evaluation efforts must still account for the fact that street medicine represents just one of many health services used by people experiencing homelessness, including mobile clinics, emergency departments (EDs), and brick-and-mortar clinics, which are delivered across multiple providers and not captured in a single electronic health record (EHR) system [16]. Each of these complexities may be heightened in the broader context of field medicine, which incorporates a wider array of services, venues, and provider types [7,17,18].

This paper describes a protocol for a mixed methods evaluation of field medicine services across Los Angeles (LA) County, California, which has the single largest unsheltered population in the nation. To address the complexities highlighted earlier, we approach this question using a “realist evaluation” framework, an approach that replaces the typical evaluator’s question of “Did it work?” with the questions “What works for whom, in what circumstances, in what respects, and how?” Our study builds on an existing monthly longitudinal survey of LA County’s homeless population that follows a cohort recruited via probability sampling throughout the county. As patients may use multiple different providers and service types, we designed a systemwide evaluation that compares outcomes for a cohort of unhoused adults who are recruited to the study via multiple field medicine providers and tracks service use across all providers. We then treat existing participants in the longitudinal survey as a comparison group, with a range of alternate observational designs described later. We also conduct qualitative interviews among field medicine patients and providers and carry out systematic observation of ride-alongs with field medicine teams. The study is embedded in a rich and multifaceted set of university and community partnerships aimed at enhancing the relevance of the study design, the wide dissemination of findings to policy and clinical audiences, and the implementation of key recommendations.

### Theoretical Framework, Research Questions, and Hypotheses

The proposed study uses a concurrent parallel mixed methods design, comprising (1) a quantitative quasi-experimental longitudinal evaluation using monthly quantitative survey data from 436 field medicine patients and 902 nonpatients, along with (2) qualitative data collected from patients and field medicine providers. Quantitative data come from the Periodic Assessment of Trajectories of Housing, Homelessness, and Health Study (PATHS), which collects monthly data from an ongoing sample of unhoused Angelenos across a wide range of relevant domains, including housing, physical and mental health status, health care usage, substance use, and food security [19]. The PATHS comparison cohort, which is broadly representative of LA County’s unhoused population, was mainly recruited during the annual “point-in-time” homeless count for 2022 to 2025. PATHS data have been widely used to address a number of issues affecting unhoused populations [19,20]. The treatment group consists of a convenience sample of field medicine patients enrolled between August 2024 and October 2025 at the point of care by a trained recruiter who enrolls patients into PATHS. We describe recruitment and statistical methods in the *Methods* section. Enrollments were conducted across a broad coalition of field medicine providers who were committed to conducting a “systemwide evaluation” as part of broader efforts to align field medicine standards, practices, and data systems. In addition to comparing the field medicine cohort to the existing PATHS comparison cohort, we will measure exposure, use, and perception of field medicine services in both groups. Qualitative data come from in-depth semistructured interviews conducted among field medicine patients, health care and social services field medicine providers, and unsheltered individuals who had not received field medicine services. Additionally, we conducted participant observations of field medicine teams, including patient recruitment strategies, patient-provider interactions (both for social and health care services), care coordination, and continuity of care.

The specific research questions (RQs) this study intends to address include the following:

- RQ1: Who accesses field medicine, and how do they differ from unhoused people who do not use field medicine?
- RQ2: How do patients use and perceive field medicine?
- RQ3: What impact does field medicine have on patients compared to nonpatients?

The study is guided by the Gelberg-Andersen Behavioral Model for Vulnerable Populations [21], which includes examining predisposing, enabling, and need factors that contribute to health behaviors (Figure 1).

**Figure 1.**
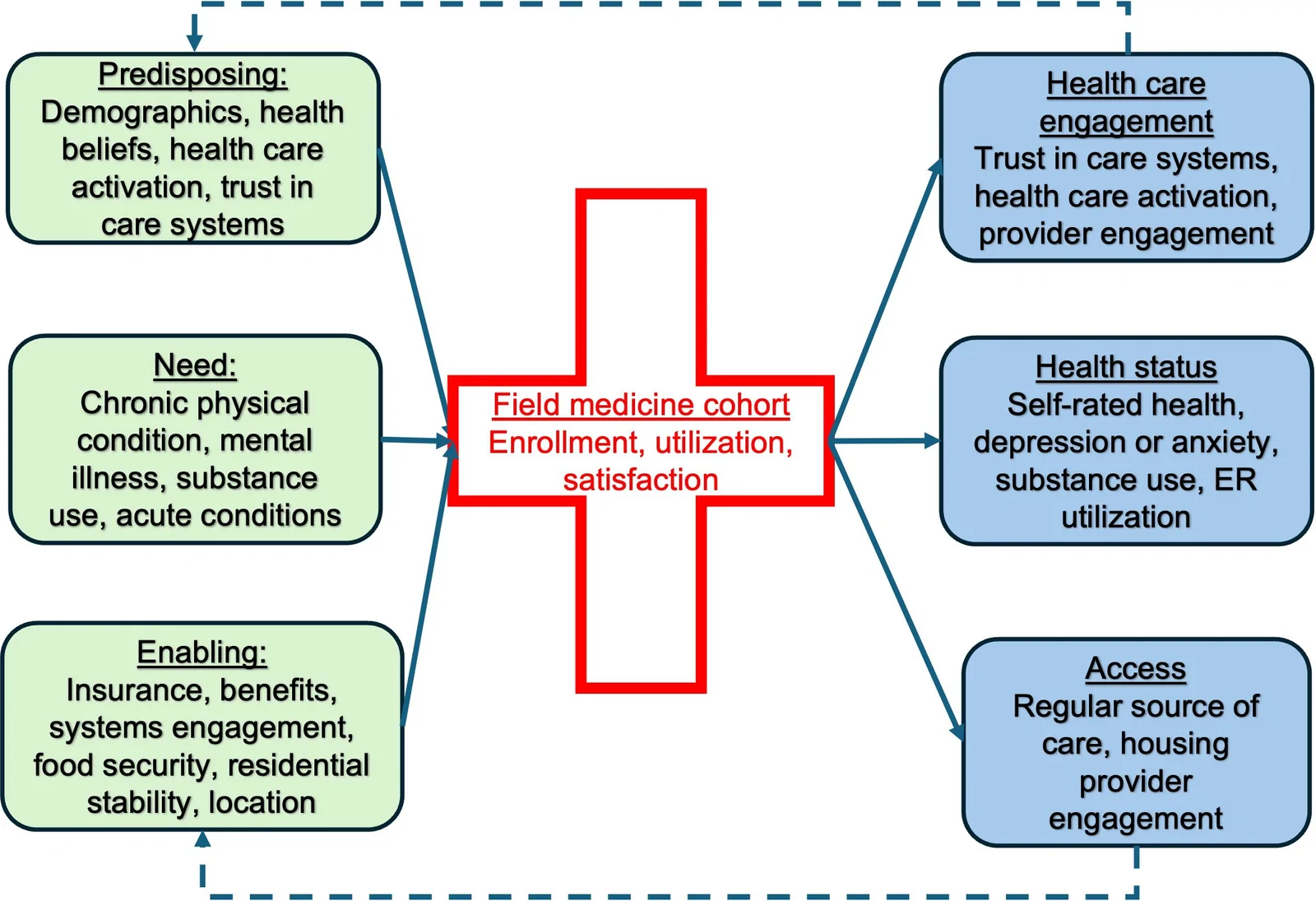
Conceptual model guiding the field medicine evaluation study. ER: emergency room.

As this is a nonrandomized evaluation, we assume that access to field medicine is determined by preexisting factors relating to predisposing, enabling, and need. This creates the potential for significant differences between patients and nonpatients at baseline, which we will test through both qualitative and quantitative analyses (RQ1). Among patients receiving field medicine services, we will assess patterns of health care usage, patient experiences, and unmet need (RQ2). As RQ1 and RQ2 are exploratory in nature, we do not offer any a priori hypotheses for these questions.

For RQ3, we will evaluate the quantitative impact of field medicine on a broad range of health outcomes and use qualitative research to interpret these findings. Importantly, field medicine carries a dual aim of improving health outcomes while also generating sustainable improvements in health care access. These indicators align with the domains identified as precursors of health in the Gelberg-Andersen model; therefore, we visualize the hypothesized pathways of field medicine impact as feeding back onto predisposing (eg, trust and activation), enabling (eg, housing and systems access), and need (eg, health status and ED use) factors. We offer the following key hypotheses:

- H3A: Field medicine exposure will be associated with improvement in factors predisposing patients to engage in further health care interaction, including health activation and trust. We note, however, that trust itself is a multidimensional construct incorporating both (1) trust in the field medicine provider versus (2) trust in the medical profession itself. Existing literature and preliminary qualitative insights suggest that field medicine may not improve trust in the broader medical profession.
- H3B: Field medicine exposure will be associated with improvement in enabling factors, including having a regular source of care, knowing how to contact the provider, access to basic needs, such as food and sanitation, and engaging with housing outreach. While most field medicine programs support housing navigation, its impact on achieved housing outcomes may be constrained by the limited availability of housing inventory in LA County.
- H3C: Exposure to field medicine will be associated with improvements in health status across a broad range of indicators. However, given the dynamic and recursive nature of health care diagnosis and treatment, access to care may simultaneously lead to an initial increase in reporting of poor health as patients become more attuned to health symptoms and concerns. For chronic conditions in particular, field medicine usage may increase the number of diagnosed conditions rather than reduce them. Similarly, while we hypothesize that better chronic disease management could reduce ED use over time, field medicine providers may also refer patients to EDs during initial interactions when unmet need is highest.

## Methods

This evaluation uses a mixed methods design, drawing on quantitative survey data from a comparative longitudinal cohort study and qualitative data from field observations and semistructured interviews. This study is reported in accordance with the STROBE (Strengthening the Reporting of Observational Studies in Epidemiology) guidelines (Checklist 1).

### Setting and Evaluation Framework

The study is situated in LA County, which has the nation’s largest unsheltered homeless population [22]. LA County also represents the country’s largest comprehensive rollout of field medicine services [23]. Over the past few years, several federally qualified health centers have introduced field medicine practices alongside smaller providers focused exclusively on the delivery of field medicine services, frequently building on the expertise of long-running programs such as those operated by Venice Family Clinic and the University of Southern California (USC) Street Medicine program. These programs frequently coordinated services and collaborated on research and training through the Community Clinic Association of Los Angeles County, which had an active Street Medicine Coordination Workgroup for over 2 years, and the ongoing California Street Medicine Collaborative. More recently, California’s Medicaid program, Medi-Cal, has increasingly funded wraparound field medicine services, such as care coordination and housing navigation through the California Advancing and Innovating Medi-Cal (CalAIM) initiative.

In July 2024, LA Care Health Plan, the country’s largest publicly operated health plan providing health insurance coverage to more than 2.4 million LA County residents, including 50,000 to 60,000 people experiencing homelessness, announced the launch of the LA County Field Medicine Program. The program is developing a countywide system to deliver primary care in field-based environments and refer patients experiencing homelessness to specialty care, care management, and other Medi-Cal–funded social services [24,25]. The academic study partners at USC and the University of California, Los Angeles (UCLA), in addition to participating in the program, have maintained ongoing communication and collaboration with this network of providers, laying the groundwork for this study.

The study goals and design emerged organically from these ongoing collaborative networks of field medicine providers. A small set of providers, including Healthcare in Action and UCLA Health Homeless Healthcare Collaborative, secured funding for program evaluations. Partners recognized the unique challenges of conducting an evaluation. As target patients are frequently displaced and have limited attachment to health care providers, it was anticipated that patients would move freely across different field medicine programs and through different brick-and-mortar clinics or EDs, making it difficult to do a stand-alone evaluation of a single provider. While each provider conducts internal analysis of EHRs, these were not considered suitable for an evaluation because (1) they lacked a comparison group, (2) EHRs lacked standardized quantitative data on patient experience, subjective health outcomes, and/or housing trajectories, and (3) EHRs are not yet interoperable, so patients cannot be tracked as they pass through multiple providers. Through conversations with academic partners, it was agreed that a survey-based evaluation would be ideal to capture a wide range of measures that could be used to track patients as they moved through the system across different providers.

A critical advantage of the survey-based approach was that a longitudinal survey already existed that included a suitable comparison group and most necessary measures. PATHS was launched in December 2021 and is carried out jointly by investigators at the UCLA Fielding School of Public Health and USC Dworak-Peck School of Social Work. PATHS data have thus far yielded scientific publications on vaccine acceptance, discrimination and violence, accelerated aging, and policing [19,20,26,27].

In January 2024, the PATHS team convened a workshop with a range of providers to establish a set of key research questions that would be feasible within the evaluation framework and of interest to a broad range of coalition members. The discussions also addressed potential limitations of the evaluation, namely, the reliance on a phone-based survey. Conversations addressed the need to pilot alternate methods to enroll patients without a phone into the study and assess differences between phone-owning patients and phone-owning nonpatients. Nevertheless, it was agreed that a comparison of phone-owning field medicine patients to a comparison group of phone-owning nonpatients would still represent a substantial leap forward in efforts to evaluate field medicine programs. Later, LA Care Health Plan joined the research consortium and provided additional funding, integration with the Field Medicine Program, and other support.

### Quantitative Research Methods

The quantitative portion of the study uses a nonequivalent control group pre-post design to compare key health outcomes among field medicine patients (ie, field medicine cohort) and nonpatients (ie, PATHS comparison cohort) at baseline and up to three 6-month follow-up intervals. Monthly surveys capture usage, patient satisfaction, and time-sensitive outcomes such as ED use. The field medicine cohort has been enrolled into the existing PATHS platform. Our primary analytic approach treats the preexisting PATHS sample as an intent-to-treat comparison cohort and acknowledges that some individuals in this cohort may have incidental exposure to field medicine services. As this contamination could bias comparisons toward the null, we outline additional analytic specifications to compare field medicine patients to nonpatients based on observed field medicine service exposure, regardless of whether patients were enrolled via the field medicine or comparison cohorts. Baseline analysis will test for selective treatment on observables (RQ1), and statistical weighting or matching techniques on observable characteristics and service area geography will be used to account for potential selection into treatment.

### Recruitment of Study Participants

The study includes 2 arms: a field medicine cohort and a comparison cohort consisting of the preexisting PATHS representative sample.

#### PATHS Comparison Cohort

The PATHS comparison cohort (hereinafter referred to as the “comparison cohort”) constitutes the comparison group for the study. We operate under the initial assumption that the representative sample will have minimal or moderate exposure to field medicine services, making it a reasonable comparison group.

Recruitment for PATHS is conducted on a rolling basis each year during data collection for the LA County Homeless Count Demographic Survey, a representative geographic sample survey conducted as part of LA County’s point-in-time homeless count. The survey is conducted from December to March each year in approximately 550 of LA County’s 2310 census tracts, with systematic canvassing of each tract. Unsheltered adults who complete the demographic survey and have a valid mobile phone number are invited to participate in PATHS. Additional recruitment has also been conducted through dedicated canvassing exercises in hotspots of unsheltered homelessness between April and July.

For those interested in participating in PATHS, surveyors either collect their phone number through a secure web form or hand out a study card with a self-enrollment option. Subsequently, prospective participants are directed to a website with study information, self-administered consent procedures, and a screening survey to determine eligibility. Eligibility requires (1) spending at least one night in the past month in a homeless shelter or unsheltered setting; (2) residence in LA County; and (3) being at least 18 years of age. Eligible participants receive a follow-up text inviting them to complete a baseline survey the next day.

On completion of the baseline survey, they are sent a survey link each month via text or email. Participants receive an electronic gift card for each survey they complete, including US $10 for the prescreen survey, US $20 for the baseline survey, US $20 for each monthly survey, and US $20 for the 6-month follow-up (ie, biannual baseline update) surveys. All surveys are conducted on a secure mobile website accessible through a phone or computer and are currently available in English or Spanish.

To define the comparison cohort for this evaluation, we include participants who had completed a baseline or 6-month follow-up survey between August 2024 and October 2025 (the period when the field medicine cohort was recruited and enrolled) and completed a monthly survey within 2 months afterward.

#### Field Medicine Cohort

The field medicine cohort was recruited through a large and representative set of field medicine providers working in LA County. To enhance the comparability of the field medicine cohort to the comparison cohort, we have replicated the recruitment methodology as much as possible. Recruitment was carried out by public health workers and case managers working with field medicine teams as well as dedicated study recruitment staff. Building on existing protocols for PATHS recruitment, the evaluation team worked with practitioners and intake staff at these providers to maximize recruitment while avoiding any disruption to service delivery or clinician rapport. Prospective participants were approached for recruitment only after they had already received field medicine services for that visit, so that study participation was not contingent on, nor perceived as a condition of, receiving care.

As in the PATHS sample, prospective participants access the prescreening questionnaire by providing their phone number or receiving a study card. A survey was developed in the Qualtrics survey software to collect phone numbers and the field medicine provider name to link a prospective participant with their enrolling provider. To ensure ease of enrollment even in settings with low wireless bandwidth, we also introduced methods to enter phone numbers of prospective participants via text message. For those enrolling via a study card, the participant can be identified with their provider based on the unique codes embedded in the study cards. Once prospective participants enter the prescreen survey, all subsequent methods for prescreen, baseline, and monthly surveys, as well as compensation schemes, are identical to those used for the PATHS comparison cohort. If a participant already captured in the existing PATHS sample is additionally enrolled via a field medicine provider (as identified by a duplicate phone number), they are migrated into the field medicine cohort.

### Quantitative Measures

Building on the Behavioral Model for Vulnerable Populations framework described earlier, we organize control and outcome measures into predisposing, enabling, and need factors that contribute to health behaviors (Figure 1). Given the strong association of housing with subsequent health outcomes and the increasing tendency for health providers to provide housing navigation, we consider housing to be a health services outcome. Field medicine services are hypothesized to positively affect predisposing and enabling factors, as described below. We hypothesize that field medicine may have more complex effects on health service need, with some services, such as ED use, expected to decline over time even as demand for more complex care needs may increase. We further hypothesize that field medicine will improve health (eg, physical and mental health). Table 1 provides a summary of PATHS variables and instruments being used to assess sample characteristics, field medicine exposure, and outcomes, and Multimedia Appendix 1 provides further details on the item(s) used to derive each variable.

Given the broad range of measures available in PATHS, we prioritize a smaller set of primary outcomes to reduce the risk of selective interpretation across the many measures. These include ED visits and hospitalizations, assessed monthly; trust in the medical profession, assessed biannually; self-rated physical health, psychological distress, and bodily pain, assessed monthly; and system navigation support, housing provider engagement, and housing status, assessed monthly. These outcomes were selected because they most directly correspond to our primary hypotheses regarding the impact of field medicine on health status, health care engagement, and trust and enabling factors of housing access, as well as their relevance and interest to program stakeholders and policymakers.

The secondary outcomes include continuity of care (medication adherence and unmet health care need) and health provider engagement; health activation; substance use behaviors and sleep quality; chronic physical disease, serious mental illness, substance use disorder, disability status, and primary health concerns; access to basic needs, including food security and sanitation resources; and access to medical care, including having a usual source of primary care. These secondary outcomes provide descriptive and exploratory context on field medicine’s potential impact and reach.

**Table 1.** Overview of Periodic Assessment of Trajectories of Housing, Homelessness, and Health Study survey measures, associated variables, and assessment schedule.

Measure	Variables and instruments	Timing
Sample characteristics		
Predisposing	Demographics (ie, age, gender identity, sexual orientation, and veteran status); social structure (ie, race or ethnicity, educational attainment, trauma, homelessness history, and criminal justice involvement); coping strategies; health care setting preference; health activation; and trust in the *medical profession*	Baseline
Need	Chronic physical disease; serious mental illness; substance use disorder; disability status; and primary health concerns	Baseline
Enabling	Health insurance coverage; receipt of government benefits (ie, Supplemental Security Income or Social Security Disability Insurance, food stamps, and unemployment); systems engagement (eg, having a usual source of primary care, contact with homeless services outreach worker, and being on a housing waitlist) Residential stability; geographic location; employment and income; access to basic needs (ie, food security and sanitation resources)	Baseline Monthly
Exposure		
Field medicine engagement	Field medicine enrollment (ie, recruited through field medicine providers); field medicine use (ie, received medical care on the street, in shelter, or from a mobile clinic); satisfaction and unmet needs with field medicine services	Monthly
Outcomes		
Primary		
Health care engagement	ED^a^ visits; hospitalizations Trust in medical profession	Biannually Monthly
Health status	Self-rated physical health; psychological distress; and bodily pain	Monthly
Access to services	System navigation support; housing provider engagement (ie, contact with homeless services outreach worker and received offers of housing); housing status (eg, ever sheltered during follow-up and months unsheltered)	Monthly
Secondary		
Health care engagement	Continuity of care (ie, medication adherence and unmet health care need); provider engagement Health activation	Monthly Biannually
Health status	Substance use behaviors; sleep quality Chronic physical disease; serious mental illness; substance use disorder; disability status; primary health concerns	Monthly Biannually
Access to services	Access to basic needs (ie, food security and sanitation resources) Access to medical care (ie, having a usual source of primary care, ability to get care for primary health concern, and ability to contact their field medicine provider)	Monthly Biannually

a ED: emergency department.

### Quantitative Analysis Methods

The data analysis and reporting will proceed in 3 aims or phases involving baseline differences (RQ1), usage and satisfaction patterns (RQ2), and health and service engagement impacts (RQ3).

#### Aim 1: Baseline Analysis

Baseline analysis will compare the field medicine cohort to the comparison cohort on the predisposing, enabling, and need characteristics identified earlier, along with demographic and geographic location controls. When comparing the field medicine vs comparison cohorts, we will report descriptive statistics as frequencies and column percentages for categorical variables and means and SDs for continuous variables. To test unadjusted associations between reported characteristics and field medicine participation, we will use the Pearson chi-square test or Fisher exact test for categorical variables where at least one expected cell count in the cross table is less than 5 and a Kruskal-Wallis rank sum test for continuous variables. To test for adjusted baseline differences, we will test logistic regression models in which the dependent variable identifies the study arm (ie, field medicine versus comparison cohort) and demographic, predisposing, enabling, and need factors are treated as independent variables. As the PATHS survey includes a “prefer not to answer” option for all questions, we anticipate a relatively high level of missingness and, thus, will conduct all analyses using first a complete case analysis followed by a complete sample using multiple imputation methods. We presume that analyses will primarily use imputed data, while complete case analysis will serve as a robustness check. Results of baseline analyses will be used for publications and policy reports that provide critical evidence on the composition of field medicine patients, populations or areas with unmet access needs for field medicine services, and potential health conditions requiring further treatment in field medicine programs. Baseline analyses will also identify the extent of baseline imbalance in the evaluation sample, thus informing the potential need for matching or other quasi-inferential strategies under aims 2 and 3.

#### Aim 2: Usage and Satisfaction Analysis

The second aim will report on patterns of field medicine service usage, knowledge, and perceptions across the 2 study cohorts, concentrating primarily on the field medicine cohort. The first focus will be on usage of field medicine services, including monthly ever-use and frequency of use across multiple months. The analysis will also explore types of services used and quantify the use of multiple field medicine providers, both for general descriptive assessment and for potentially informing heterogeneity analysis. This analysis will inform the impact evaluation in aim 3 in a variety of ways. First, we will quantify the extent of field medicine usage in the comparison cohort, quantifying potential contamination in the design and informing alternative uptake-based designs. Second, we will explore the frequency of service use, which will be used for descriptive purposes and for the construction of dose variables indicating frequency of field medicine contact. We will characterize the extent to which patients enrolled through a particular field medicine provider also obtained services from other field medicine providers. Finally, we will measure patient satisfaction with the services received each month. In all cases, these data will provide helpful policy-relevant descriptions of usage profiles and patient engagement. We will also conduct scientific analysis of variations in patient retention, usage, and progression of intensity of service engagement.

#### Aim 3: Impact Evaluation

The final aim of the study is to evaluate the impact of field medicine services on patient health outcomes; access to health, social, and housing services; and trust in the medical profession in comparison to the comparison cohort. Building on the realist evaluation approach, we will use a variety of ways of identifying “exposure” to field medicine services as the treatment measure. The primary models will use an intent-to-treat approach, comparing individuals enrolled in the field medicine cohort to those in the comparison cohort. Alternate design will instead use a usage-based independent variable, including ever-use or frequency or cadence of use of field medicine services, thereby accounting for the fact that there may be crossover between the study arms, with some members of the comparison cohort using field medicine services more heavily than treated individuals. Additional models will disaggregate overall field medicine treatment effects into effects associated with specific providers or types of services. The analysis will include a mix of outcome measures captured either monthly or at specific 6-month (biannual) intervals (January and July of each calendar year). We describe separate statistical estimation strategies for each.

#### Biannual Measures

Two-sample, 2-tailed *t* tests will be used to determine whether the mean change in each outcome from baseline to follow-up is significantly different between the field medicine and comparison cohorts. Mixed effects models for repeated measures will assess whether the field medicine cohort shows statistically significant improvements in health care engagement, health status, and access to services at each biannual follow-up compared to the comparison cohort. All models will adjust for potential baseline differences in primary outcomes across study groups.

An advantage of repeated-measures analysis is that participants with baseline data who are missing at follow-up can still be included in the analysis. To account for the rolling recruitment of participants over a 12-month period, we will adjust models for the enrollment month. Period fixed effects will also be included to address any changes in the policy environment over the follow-up period. All analyses will be conducted in Stata software (version 19; StataCorp LLC) and R software (version 4.1.2; R Foundation for Statistical Computing), with appropriate weighting to account for our sample selection strategy. We will assess clustering of variance within sample clusters by examining the intraclass correlation and, if necessary, correct SEs for multivariate results using appropriate clustering correction methods.

#### Monthly Measures

Monthly measures may display considerable variation within person as well as distinct trajectories for different population groups. For these models, we will use hierarchical generalized linear models (HGLMs) that can be used to estimate a program effect in the presence of correlated errors. HGLM is well suited for modeling intraindividual changes in outcomes and interindividual differences in these changes, as well as for handling unbalanced data structures and missing within-person data. A series of 2-level HGLMs, in which monthly observations (level 1) are nested within individuals (level 2), will be used to examine the effects of field medicine on temporal changes in monthly health measures within individuals over time, controlling for any time-varying covariates and time-invariant baseline covariates (ie, sociodemographic characteristics, duration of homelessness, and coping strategies). We will include fixed and random effects for the intercept and slope for time to examine the mean initial status and rate of change, individual deviations from these means, and whether the field medicine intervention impacts initial status and rate of change.

Level 1 (within-person level):

$Y_{it}=\beta_{0i}+\beta_{1i}Time_{it}+\beta_{2i}(TVC_{it}-{\bar{TVC}}_{i})+e_{it}$

Level 2 (between-person level):

$\begin{array}{rl} \beta_{0i} & =\gamma_{00}+\gamma_{01}MED+\gamma_{02}\left({\bar{TVC}}_{i}\right)+\gamma_{03}TIC_{i}+r_{0i}\\ \beta_{1i} & =\gamma_{10}+\gamma_{11}MED+r_{1i}\\ \beta_{2i} & =\gamma_{20} \end{array}$

where *i* is the index of persons, *t* is the index of time per month, MED is intervention assignment, $\bar{TVC_{i}}$ represents the mean value of time-varying covariates for an individual across all time points (ie, the person-specific mean), and$\left(TVC_{ij}-\bar{TVC_{i}}\right)$ represents the time-specific deviation of the variable from the person-specific mean. β_0*i*_ and β_1*i*_ represent the intercept (ie, initial status of the outcome) and slope (ie, rate of change) for individual *i*, γ_00_ and γ_10_ are the overall mean intercept and slope, and *r*_0*i*_ and *r*_1*i*_ are the individual-specific deviations from the mean intercept and slope (random effects).

#### Statistical Adjustments for Nonrandom Assignment

To improve our confidence in the inference emerging from our analyses, we will use matching and weighting methods to account for key threats to validity, including (1) differential selection into field medicine services based on prior health and other factors, which would limit our ability to compare field medicine patients and nonpatients; and (2) selective attrition from the cohort over time, which may also potentially differ between field medicine patients and nonpatients.

Our primary approach for addressing selection on observables will be matching. We will match field medicine cohort members to comparison cohort members on demographic covariates (eg, age, gender identity, and race or ethnicity), geographic location at recruitment, baseline health status (eg, chronic physical disease, serious mental illness, substance use disorder, and patient health activation), and service access (eg, receiving government benefits and housing systems engagement). We will assess covariate balance before and after matching with standard diagnostic approaches (eg, visual plots, standardized mean differences, and variance ratios); outcome analyses described earlier (biannual and monthly models) will then be conducted within the matched sample. As a secondary and sensitivity analysis, we will construct inverse probability of treatment weights using the same set of covariates, estimated via a propensity score model predicting field medicine cohort membership. This approach retains the full analytic sample rather than restricting to matched pairs. Consistency across matching- and weighting-based approaches will strengthen our confidence in the robustness of findings; discrepancies will be reported and interpreted as a limitation of the nonrandomized design.

We note that because the field medicine cohort is enrolled directly into the existing PATHS platform and follows identical recruitment, consent, and survey procedures as the comparison cohort, both cohorts share the same requirement of phone access and willingness to complete a phone-based survey. This shared enrollment pipeline reduces differential selection on these dimensions between the 2 study arms. We discuss the implications of phone-based enrollment for the generalizability of the study population as a whole in the *Discussion* section.

Given the preliminary nature of this study, we may find that study retention is an important outcome in and of itself, due to the potential for differential attrition across cohorts. The importance of these selection-adjusted models will depend in large part on the extent of observable baseline differences between the cohorts. Regardless of the balance achieved on observables, our nonequivalent control group design may also introduce potential unobserved variation in the samples. Depending on the extent of this concern, we will use geographic data on the service coverage areas of field medicine providers to exogenously identify selection into field medicine service access. Additionally, we will conduct sensitivity analyses for unmeasured confounding, including tipping point analyses (E-values and robustness values) and benchmarking against observed covariates within an omitted-variable-bias framework, to quantify how strong an unobserved confounder would need to be to alter our substantive conclusions [28,29].

### Qualitative Research Methods: Recruitment of Study Participants

#### Qualitative Participant Observation

For the qualitative component of the study, we conducted qualitative interviews and participant observation with 9 field medicine teams. These teams are affiliated with 3 distinct field medicine programs. Program directors facilitated recruitment by sharing the contact information of team leads who were likely to be interested in participating. IDMN then contacted those leads directly to invite participation. Most teams operate on the west side of LA County, although some fieldwork has also been conducted in the southern region of the county.

#### Qualitative Semistructured Interviews

IDMN directly recruited field medicine providers, including physicians, physician assistants, nurse practitioners, nurses, and community health workers (CHWs), for semistructured interviews during participant observation activities. Field medicine patients and unhoused individuals located within the service areas of participating teams but not currently receiving care were recruited for semistructured interviews using 2 recruitment strategies. First, field medicine providers directly referred potential participants—both patients and nonpatients—after inviting them to participate in the study during routine outreach. Providers were encouraged to ensure demographic variation across gender, race, and ethnicity. Once a potential participant agreed to participate in the interview, the field medicine provider introduced IDMN, who would conduct the interview, and then stepped away to allow the interview to proceed privately. If IDMN was not present in the field when a potential participant agreed to participate, the field medicine provider scheduled the interview for a date and time convenient for the participant, accompanied IDMN to the scheduled appointment, introduced her to the participant, and likewise stepped away before the interview began. Second, IDMN conducted in-person recruitment by accompanying previously shadowed teams during street outreach and personally inviting patients and nonpatients to participate in the study. When potential participants agreed to participate, IDMN conducted the interview immediately following recruitment.

### Qualitative Measures

#### Qualitative Participant Observation

Participant observation was used to examine the everyday practices of field medicine teams and to gain a deeper understanding of how care is delivered in situ. The primary focus is on the processes through which patients are identified and recruited, the nature of provider-patient interactions, and how clinical assessments and service offerings unfold in real time. Attention is given to how teams address both medical and social needs, navigate barriers to continuity of care (ie, scheduling follow-up visits and making specialty referrals), and coordinate with other service providers. We also closely observe how trust is built between providers and unhoused patients.

#### Qualitative Semistructured Interviews

Semistructured interviews with patients focused on their experiences with field medicine, including how they initially connected with providers, barriers to accessing care regularly, services received, unmet needs, aspects of care they valued, and suggestions for improvement. Interviews with nonpatients explored their current health care needs, patterns of health care usage, knowledge of field medicine services, and perceived barriers to accessing field medicine. Providers were asked about perceived barriers and facilitators to delivering health care on the streets and ensuring continuity of care, strategies for navigating those challenges, the scope of medical and social services they offer, and gaps in care delivery. Interviews ranged from 30 to 90 minutes and were conducted in person for the unhoused subsample and either in person or by phone for providers. All interviews were audio recorded with participant consent. Unhoused participants received a US $40 gift card as a token of appreciation for their time.

#### Qualitative Analysis Methods

Semistructured interview transcripts and field notes were imported into NVivo (version 15; Lumivero) for analysis. We used a thematic analysis [30], with codes developed both deductively—based on the study’s core questions (ie, experiences with field medicine, access barriers and facilitators, continuity of care, satisfaction, and unmet needs)—and inductively, through close coding of the interview transcripts and field notes to capture emergent themes. IDMN and a graduate research assistant coded the same 2 interview transcripts separately and then engaged in an iterative process of refining the codes—merging, expanding, and collapsing them—until they reached consensus on the final codebook. Once the codebook was finalized, the remaining interview transcripts were coded independently by IDMN and the graduate research assistant using NVivo software. The same software was used by IDMN and 2 undergraduate research assistants to code the field notes.

We use both methodological and data source triangulation to enhance the depth of our qualitative findings. Methodologically, we integrate data from semistructured interviews and participant observation. Interviews capture participants’ perspectives, experiences, and perceptions related to field medicine, while participant observation provides insight into real-time provider-patient interactions, care delivery processes, and the broader context in which services were delivered. Observational data will help complement, clarify, or complicate interview accounts, allowing us to identify gaps between reported and enacted practices and uncover patterns that are not always explicitly described by participants. In addition, we use data source triangulation by including multiple participant groups—field medicine patients, nonpatients, and providers—which allows us to examine the same phenomena from diverse viewpoints. This combined triangulation strategy strengthens our analysis and will lead to a more comprehensive understanding of the implementation and lived experience of field medicine care.

#### Mixed Methods Convergence

##### Overview

As appropriate, qualitative and quantitative results will be integrated and reconciled to achieve several different functions. First, reconciled results will be used to identify convergences, areas where qualitative data can confirm quantitative findings, and vice versa. Second, the research team will use qualitative data complementarily to provide depth and understanding to findings that emerge from quantitative analyses [31].

Although the study will use a parallel mixed methods design, the strong provider partnership will afford numerous opportunities to ensure convergence and triangulation between the results of the quantitative and qualitative research. We have explicitly developed the study aims and methods to ensure that quantitative and qualitative research methods speak to each of the key study questions, as detailed in the following sections.

##### Research Question 1

The quantitative arm will compare the field medicine cohort with the comparison cohort across predisposing, enabling, and need factors, sociodemographic characteristics, and geographic location. The qualitative interviews provide depth to the predisposing, enabling, and need factors found in the survey for both field medicine patients and nonpatients. The qualitative interviews and participant observations will also elucidate additional factors contributing to people’s access to field medicine that were not necessarily present in the quantitative analysis.

##### Research Question 2

Both quantitative and qualitative components of the study will identify the services provided in field medicine. For the services salient in both study components, the qualitative findings will add depth by illustrating how those services are provided. The quantitative analysis will also measure the patterns of field medicine usage among patients. The qualitative findings will help explain those patterns by describing the factors that specifically affect continuity of care in field medicine. The quantitative analysis will measure the knowledge and perceptions of both patients and nonpatients regarding field medicine. The interviews will provide depth to both the positive and negative perceptions present in the survey, as well as perceptions not captured in the survey. Finally, the participant observations will illustrate the aspects of clinician-patient interactions that have been identified as either positive or negative by interviewed participants from the patient group.

##### Research Question 3

The quantitative component will measure self-rated health outcomes of patients, and the interviews will explore patients’ perceived impact of field medicine on their well-being. These 2 sources of information will complement each other, providing a broader understanding of how field medicine contributes to perceived health. The survey will also measure access to health, social, and housing services among the field medicine cohort compared to those of the comparison cohort. The qualitative component will help understand why field medicine patients might not have significantly better access to health, social, and housing services by analyzing the barriers and facilitators to housing and specialty care observed in the field and reported by patients and providers. Finally, the survey will measure trust in the medical profession among field medicine patients and nonpatients. Interviews with field medicine patients will provide insights into why trust might not differ significantly between cohorts.

Table 2 further summarizes the specific quantitative measures and qualitative themes that will be used to address each research question.

We will achieve further mixed methods convergence through the deep feedback networks associated with the project and described in the *Setting and Evaluation Framework* section. The study’s provider and academic partners convene regularly through the Homelessness Policy Research Institute housed at USC, through the California Street Medicine Collaborative, and through the LA Care health network. Each of these events will provide regular opportunities to present qualitative and quantitative findings to providers and individuals with lived expertise on homelessness, thereby allowing us to reconcile findings across methods. The lead field medicine partner agencies also meet with members of the research team for more intensive day-long meetings once or twice annually.

**Table 2.** Summary of research questions mapped to corresponding quantitative measures and qualitative themes.

Research questions	Quantitative measures	Qualitative themes
Are those PEH^a^ who engage with field medicine providers different from a comparison cohort of unsheltered individuals in LA County who do not engage with field medicine?	Comparison of field medicine cohort versus comparison cohort on predisposing, enabling, and need factors, along with sociodemographic characteristics and geographic location.	Intake procedures observed and described by providers and patients; predisposing, enabling, and need factors described by patients and nonpatients.
For those engaged in field medicine, how extensively do participants use field medicine services, what kind of services do they use, and are they satisfied with those services?	Patterns of field medicine service use, knowledge, and perception across the 2 study groups.	Observed and reported health and social services provided to field medicine patients; observed and reported barriers to continuity of care (multiple use or visits); patients’ perceptions of field medicine services; patients’ reported unmet needs; and observed patient-clinician interactions.
When compared to individuals of initially comparable health, do individuals who use field medicine report improvements in health status, systems engagement, housing, and other critical outcomes?	Patient health outcomes, access to health, social, and housing services, and trust in the medical profession among the field medicine cohort versus the comparison cohort.	Patients’ perceived impact of field medicine on their well-being; and observed and reported barriers and facilitators to housing referrals and specialty care referrals.

a PEH: people experiencing homelessness.

### Ethical Considerations

#### Ethics Approval

This study was approved by the UCLA South General Institutional Review Board and UCLA North General Institutional Review Board (protocol IRB21-001148 and IRB-22‐1985, respectively).

#### Consent Procedures

Participants in both the field medicine and comparison cohorts complete self-administered informed consent prior to screening, as described earlier.

For field observations, IDMN obtained oral informed consent directly from field medicine providers before observations and from patients before observing their clinical encounters. For semistructured interviews, IDMN obtained oral informed consent directly from each participant before the interview.

#### Data Privacy and Distress Safeguards

Survey participants are assigned a unique study identifier upon screening, independent of personally identifiable information (PII), to deidentify their study data. Although participant contact information (eg, phone numbers and email addresses) is collected during recruitment and enrollment, this information is used only to distribute subsequent study surveys, maintain contact with participants during the study period, and distribute financial incentives. Contact information is stored only on the secure data collection platform and is stripped from survey responses prior to transfer to the secure, encrypted server used for data storage and analysis. Only study team members with authorized access permissions have access to PII. As survey responses are deidentified from contact information prior to analysis, participant confidentiality is maintained during dissemination of study findings in papers, presentations, and reports.

Surveys are self-administered and completed remotely on participants’ own schedules via a secure mobile website; participants are not required to be in any particular physical location to complete them. As part of our trauma-informed survey design, to reduce distress and response burden while answering monthly surveys, all questions in the surveys include a “prefer not to answer” option, allowing participants to decline to respond to any question without penalty or exclusion from the study.

#### Data Sharing

Deidentified quantitative survey data will be made available in a public data repository (eg, the Inter-university Consortium for Political and Social Research) following study completion, consistent with data sharing requirements of the study’s funders. Only deidentified data, stripped of PII as described earlier, will be shared. The publicly shared dataset will not include geographic identifiers. Geographic data will be governed by a restricted data use policy and made available only upon request through a data use agreement with the study team.

## Results

This study is funded from January 2024 to June 2027.

### Quantitative Results to Date

#### PATHS Comparison Cohort

Between December 2021 and October 2025, PATHS screened 3004 respondents, of whom 1836 (61%) were eligible and completed a baseline survey. Among these, 1413 (77%) proceeded to complete at least one monthly survey and thus had been officially enrolled in the study since study launch. For this evaluation study, the comparison cohort will include 902 participants who had completed a baseline or 6-month follow-up survey between August 2024 and October 2025 (the period when the field medicine cohort was recruited and enrolled) and completed a monthly survey within 2 months afterward.

#### Field Medicine Cohort

Recruitment of the field medicine cohort began in August 2024 and concluded in October 2025. During this period, we partnered with 6 field medicine providers that operate in LA County, including Healthcare in Action, Venice Family Clinic, UCLA Health Homeless Healthcare Collaborative, Clínica Romero, Bartz-Altadonna Community Health Center, and the Los Angeles County Department of Health Services. Patients were recruited through their field medicine services operations, which included mobile clinics, backpack medicine teams, static sites at parks or community centers, encampment-based care, and shelter-based or transitional housing clinics.

As of October 2025, 847 field medicine patients had been referred from our field medicine partners and screened for eligibility, with 749 (88%) meeting eligibility criteria. Of these, 542 (72%) completed the baseline survey, and 436 (80%) participants completed a subsequent monthly survey, officially enrolling in the study.

Recruitment efforts were strengthened by the development of strong, collaborative relationships with field medicine teams, whose support was instrumental in engaging potential participants and facilitating enrollment across a wide range of outreach settings. These partnerships enabled flexible, real-time coordination and helped build trust with patients receiving care in nontraditional environments.

At the same time, the study team encountered numerous operational challenges that required ongoing adaptation. Participant turnout at street-based sites was inherently unpredictable and influenced by a range of contextual factors, such as weather, safety concerns, and competing priorities faced by patients. Recruitment was further impacted by 2 significant period effects occurring within LA County: widespread wildfires in January 2025 and a wave of roaming US Immigration and Customs Enforcement (ICE) raids targeting public spaces, which were followed by civil unrest in June 2025. These events intensified law enforcement presence and fostered a climate of fear, unease, and instability in the unhoused population.

In response, the team implemented a range of adaptive strategies to maintain study continuity and protect all involved. These included shifting outreach to safer locations, modifying schedules in real time, implementing a buddy system for data collection, and strengthening communication protocols. Identifying safer locations involved monitoring local independent news sources to identify areas of LA County with ICE activity and allowing teams to determine potential areas to avoid. It was through consistent monitoring of these various sources that, for example, we became aware of an ICE raid at MacArthur Park, which disrupted scheduled field medicine services on July 7, 2025. Daily schedule adjustments were communicated through work phones and a designated team group chat to ensure timely coordination. We implemented a buddy system for data collection as well, so that together they could use situational awareness to assess the different sites we visited. Throughout this period, we also maintained open communication with service providers and partners, following their lead in identifying safety risks and adapting to site-specific procedures as needed. During law enforcement events, all in-person recruitment activities were paused; any participant present at the time was informed of the situation and prioritized for immediate safety. Recruitment activities only resumed once participants had moved to a safe location alongside staff and remained willing to continue. To further minimize risk to participants, the study does not collect any information on immigration status, and survey data are stored without directly identifying information (see *Data Privacy and Distress Safeguards* section).

### Qualitative Results to Date

#### Participant Observation

Between October 2022 and October 2024, more than 300 patient-clinician interactions were observed during a total of 82 field visits, resulting in approximately 500 hours of participant observation. IDMN joined field medicine teams at the beginning of their shifts and accompanied them throughout their outreach rounds—typically 6 hours—by riding along and observing their interactions with patients, colleagues, and partner organizations. Team composition varied: smaller teams typically included a health care provider (eg, physician, physician assistant, nurse, or nurse practitioner) and a CHW, while larger teams might also include a medical assistant and security officer. Teams were diverse in terms of age, gender, race, and ethnicity.

Participant observation took place at various locations, including medical vans, sidewalks, parking lots, beaches, parks, and interim sites (eg, shelters, board and care facilities, and motels). During each shift, IDMN took notes on a mobile phone to capture key moments, dialogues, and reflections. That same day, these notes were expanded into detailed, narrative-style field notes that captured the flow of the day and contextualized the observations.

#### Qualitative Semistructured Interviews

Between May 2024 and October 2024, IDMN interviewed 15 field medicine providers—including physicians, physician assistants, nurse practitioners, nurses, and CHWs—as well as 23 field medicine patients and 12 unhoused individuals located within participating service areas but not currently receiving care. The final sample size was determined by thematic saturation rather than a predetermined numerical target, with recruitment concluding once additional interviews no longer generated novel themes or perspectives [32].

### Timeline

Survey data collection for the evaluation, spanning both the field medicine and comparison cohorts, began in August 2024 and is projected to continue through April 2027, allowing for a minimum of 18 months of study participation per participant. Baseline analysis (aim 1) is being conducted between January 2026 and August 2026, and evaluation analyses (aims 2 and 3) are expected to occur from November 2026 through June 2027. Field notes and interview transcripts with patients and nonpatients have been fully coded. Half of the interviews with providers are coded, and the rest will be coded by winter 2027. Findings from aim 1 (baseline analysis) are expected to be submitted for publication in fall 2026, and findings from aims 2 and 3 (impact evaluation) are expected to be submitted for publication in fall 2027.

## Discussion

### Overview

The implementation of this study comes during a changing landscape of approaches to unsheltered homelessness. There have simultaneously been increased calls to further criminalize homelessness and to provide more immediate access to shelter and housing. Unsheltered people also face growing challenges posed by recurrent disasters, climate change, and other pervasive threats. Within this increasingly challenging landscape, field medicine programs offer the potential to buffer people experiencing homelessness against persistent health-related risks and improve prospects for gaining access to housing. There have been widespread calls for more rigorous evidence on the impacts of field medicine programs to contribute to standards of practice, and this study will fill this gap [7].

### Strengths

This study will thus be critical in providing the first rigorous mixed methods evidence on the potential impacts of field medicine programs on multiple dimensions of patient well-being and systems engagement. The study applies the widely used Behavioral Model for Vulnerable Populations, incorporating factors driving health systems engagement as both controls and outcomes. The study uses (1) a large and trauma-informed quantitative survey among more than 1300 participants and a wide range of validated measures; (2) qualitative interviews and participant observations with providers, patients, and nonpatients; and (3) mixed methods reconciliation of findings within the behavioral model. By incorporating the predisposing, enabling, and need factors included in the behavioral model as both predictors of access and as study outcomes, this study can also contribute to the design of future studies that explicitly target barriers to health care access in a systematic fashion.

Findings will be shared widely through scientific publications, conference presentations, workshops with networks of field medicine providers, and policy briefs.

### Anticipated Findings

We anticipate that the evaluation will produce actionable results on the impact of field medicine programs across the program cycle, from usage to satisfaction with health and health care usage. In keeping with the realist evaluation framework and our mixed methods approach, we do not necessarily prioritize any specific hypotheses, quantitative versus qualitative findings, or particular quantitative designs. As noted in the *Theoretical Framework*, *Research Questions*, *and Hypotheses* section, many of the primary outcome variables may be subject to multiple contradictory mechanisms. We noted the particular example of ED use, which could rise in the short term due to referrals by field medicine providers but decline in the longer term due to better engagement and condition management. While this particular example might yield specific predictions on potential changes in ED usage over time or with increasing dosage of field medicine treatment, many other contradictory effects may be more nuanced. Given these parameters, we anticipate that the most impactful findings of the study will answer questions surrounding when and how field medicine can be beneficial, rather than whether it is beneficial at all. We also note the complexity of quantitative design itself and of the mixed methods approach. Even any confirmations of specific hypotheses would need to be corroborated in unadjusted and adjusted models, in models using field medicine exposure versus dose-response, and in matched or weighted models. These quantitative findings would need further corroboration and elaboration in qualitative data. Finally, all results would be subject to extensive confirmation and review with the study’s health care provider and insurer partners, with broader field medicine networks, and with people with lived expertise.

### Limitations

This evaluation has a few limitations. First, although we will use matching and weighting on observed baseline demographic, geographic, and health status covariates to address selection on observables, as a nonrandomized study, unmeasured confounding in comparisons between the field medicine and comparison cohorts may bias findings. Second, both cohorts are enrolled through the same phone-based recruitment pipeline, which excludes unhoused individuals without reliable phone access or willingness to engage in a phone-based survey. While this shared requirement reduces differential selection between the 2 cohorts specifically, it may exclude participants who are most disconnected and vulnerable, thus limiting the generalizability of study findings to the broader population of people experiencing homelessness in LA County. Future studies could consider alternative enrollment strategies and longitudinal data collection to better reach populations without reliable phone access. Third, we anticipate contamination between study arms, as members of the comparison cohort may have incidental exposure to field medicine services over the course of the study. Although we account for this through usage-based exposure measures in aim 3, any residual contamination would be expected to bias intent-to-treat estimates toward the null, meaning true effects of field medicine may be somewhat larger than observed. Fourth, the study relies on monthly and biannual self-reported measures of health status, health care engagement, and service access, which are subject to social desirability and recall bias and cannot be independently verified against clinical records. Relatedly, the monthly survey burden may contribute to selective attrition among participants with greater instability, which could differ systematically between cohorts. Finally, findings are drawn from a single county with a large-scale and coordinated field medicine infrastructure; therefore, results may not generalize to jurisdictions with smaller, less coordinated field medicine programs.

## Supplementary material

10.2196/94248 Multimedia Appendix 1 Periodic Assessment of Trajectories of Housing, Homelessness, and Health Survey (PATHS) measures, variables, and assessment schedule for evaluation.

10.2196/94248 Checklist 1 STROBE checklist.

## Abbreviations

CalAIM: California Advancing and Innovating Medi-Cal
CHW: community health worker
ED: emergency department
EHR: electronic health record
HGLM: hierarchical generalized linear model
ICE: Immigration and Customs Enforcement
LA: Los Angeles
PATHS: Periodic Assessment of Trajectories of Housing, Homelessness, and Health Study
PII: personally identifiable information
RQ: research question
STROBE: Strengthening the Reporting of Observational Studies in Epidemiology
UCLA: University of California, Los Angeles
USC: University of Southern California

## References

[1] Roncarati JS, Baggett TP, O’Connell JJ (2018). Mortality among unsheltered homeless adults in Boston, Massachusetts, 2000-2009. JAMA Intern Med.

[2] Morrison DS (2009). Homelessness as an independent risk factor for mortality: results from a retrospective cohort study. Int J Epidemiol.

[3] Brown RT, Hemati K, Riley ED (2017). Geriatric conditions in a population-based sample of older homeless adults. Gerontologist.

[4] Richards J, Kuhn R (2023). Unsheltered homelessness and health: a literature review. AJPM Focus.

[5] Su KY, Feldman BJ, Feldman CT, Saluja S, Coulourides Kogan AM, Cousineau MR (2024). Behavioral health care delivery through street medicine programs in California. Community Ment Health J.

[6] Stefanowicz M, Feldman B, Robinson J (2021). House calls without walls: street medicine delivers primary care to unsheltered persons experiencing homelessness. Ann Fam Med.

[7] Schwan KJ, Feldman CT, Feldman BJ (2026). Advancing street medicine - aligning research, implementation, and policy for impact. NEJM Catal Innov Care Deliv.

[8] Lynch KA, Harris T, Jain SH, Hochman M (2022). The case for mobile “street medicine” for patients experiencing homelessness. J Gen Intern Med.

[9] Enich M, Tiderington E, Ure A (2023). Street medicine: a scoping review of program elements. IJOH.

[10] Medellin T, Moczygemba LR, Thurman W (2024). A qualitative study to describe the nature and scope of street medicine programs in the United States. Int J Environ Res Public Health.

[11] Kaufman RA, Mallick M, Louis JT, Williams M, Oriol N (2024). The role of street medicine and mobile clinics for persons experiencing homelessness: a scoping review. Int J Environ Res Public Health.

[12] Takada S, Dawson MM, Chung UYR (2026). Hypertension control among patients receiving care from a field-based homeless healthcare team. J GEN INTERN MED.

[13] Wallace CR, King J, Nichols T (2026). Buprenorphine initiation by a street medicine program is associated with reduced opioid-related emergency department utilization among people experiencing homelessness. Drug Alcohol Depend.

[14] Natsoulis A, Nussbaum C, Howard L (2026). Street-based hepatitis C treatment and patient experiences among adults experiencing unsheltered homelessness. JAMA Netw Open.

[15] Saluja S, Jembere N, Cousineau M, Directo MP, Riggs RV, Isonaka SK (2026). Evaluation of a street medicine based post-discharge care program for patients experiencing homelessness. Healthc (Amst).

[16] (2024). Place of service code ‘27’ may be used for street medicine. California Department of Health Care Services.

[17] Cook A, Nuti SV, Johnson AK, Lan Y, Jacobson L, Long T (2026). Operating a street medicine program within New York City’s public health care system. NEJM Catal Innov Care Deliv.

[18] Simenz CJ, Marchlewski A, Nelson DA (2026). Step-by-step: the BRIDGE framework for equitable outreach to people experiencing homelessness. Health Promot Pract.

[19] Kuhn R, Chien J, Guzman Hernandez N (2025). Periodic Assessment of Trajectories of Housing, Homelessness, and Health Study (PATHS): protocol for a prospective cohort study of people experiencing homelessness. JMIR Res Protoc.

[20] Chien J, Henwood BF, Kuhn R (2026). Criminalizing homelessness: longitudinal associations of police encounters and homeless sweeps with psychosocial health among the unhoused community in Los Angeles. Social Science & Medicine.

[21] Gelberg L, Andersen RM, Leake BD (2000). The Behavioral Model for Vulnerable Populations: application to medical care use and outcomes for homeless people. Health Serv Res.

[22] Henry M, Sousa T, Roddey C, Gayen S, Bednar T (2020). The 2020 Annual Homeless Assessment Report (AHAR) to Congress. https://www.huduser.gov/portal/sites/default/files/pdf/2020-ahar-part-1.pdf.

[23] Feldman B, Feldman CT, Kogan C, Saluja S, Cousineau MR (2023). The California street medicine landscape survey and report. https://www.chcf.org/wp-content/uploads/2023/03/CAStreetMedLandscapeSurveyReport.pdf.

[24] (2025). LA County Field Medicine Program overview: LAC-FMP-004. LA Care Health Plan.

[25] (2024). LA Care and Health Net commit $90 million to launch two innovative health care programs to support those experiencing homelessness in LA county. L.A. Care Health Plan.

[26] Padwa H, Chien J, Henwood BF, Cousins SJ, Zakher E, Kuhn R (2024). Homelessness, discrimination, and violent victimization in Los Angeles County. Am J Prev Med.

[27] Kuhn R, Henwood B, Lawton A (2021). COVID-19 vaccine access and attitudes among people experiencing homelessness from pilot mobile phone survey in Los Angeles, CA. PLoS One.

[28] Cinelli C, Hazlett C (2020). Making sense of sensitivity: extending omitted variable bias. Journal of the Royal Statistical Society Series B.

[29] Faries D, Gao C, Zhang X (2025). Real effect or bias? Good practices for evaluating the robustness of evidence from comparative observational studies through quantitative sensitivity analysis for unmeasured confounding. Pharm Stat.

[30] Braun V, Clarke V (2006). Using thematic analysis in psychology. Qual Res Psychol.

[31] Palinkas LA, Mendon SJ, Hamilton AB (2019). Innovations in mixed methods evaluations. Annu Rev Public Health.

[32] Hennink MM, Kaiser BN, Marconi VC (2017). Code saturation versus meaning saturation: how many interviews are enough?. Qual Health Res.

