# Supplementary material for: Systemwide Evaluation of Field Medicine Programs in Los Angeles County: Protocol for a Mixed Methods Longitudinal Observational Study
Source: JMIR Res Protoc. 2026 Oct 14;15:e94248. doi: 10.2196/94248 (PMC13664992; doi:10.2196/94248)
Supplement: Supplementary file 1 [file resprot-v15-e94248-s001.docx]

**Periodic Assessment of Trajectories of Housing, Homelessness and Health Survey (PATHS) measures, variables, and assessment schedule for evaluation**

| **Dimension** | **Measure** | **Survey** | **Source** | **Items** |
| --- | --- | --- | --- | --- |
| Predisposing | Age (Year of Birth) | Baseline | Team | What is your year of birth? |
| Predisposing | Sex/Gender identity | Baseline | Team | With which sex/gender do you most identify?   a. Male b. Female c. I do not identify as male or female.   [If “I do not identify as male or female”]  Please share which of the following best reflects your gender identity:   a. Trans (M2F) b. Trans (F2M) c. Gender non-binary, assigned female at birth d. Gender non-binary, assigned male at birth e. Intersex f. None of the above |
| Predisposing | Sexual orientation | Baseline | Team | Do you consider yourself to be:   a. Heterosexual or straight b. Homosexual c. Bisexual d. Other |
| Predisposing | Racial identity | Baseline | Team | What best describes your race? (Select all that apply)   a. Black/African American b. White c. Asian d. Native Hawaiian or Pacific Islander e. American Indian/Alaskan Native f. Another race |
| Predisposing | Ethnicity | Baseline | Team | Do you consider yourself to be Hispanic or Latino/a?   a. Hispanic or Latino/a b. Not Hispanic or Latino/a |
| Predisposing | Veteran status | Baseline | Team | Have you served in the US military?   a. Yes b. No |
| Predisposing | Educational attainment | Baseline | Team | What is the highest level of education you received?   a. 8^th^ grade or less b. Some high school c. High school or GED d. Some college, no degree e. Associates degree or vocational certificate f. Bachelor’s degree or higher |
| Predisposing | Trauma | Baseline & Biannual | PC-PTSD-5 | Sometimes things happen to people that are unusually or especially frightening, horrible, or traumatic.  Have you ever experienced this kind of event?   a. Yes b. No   [If “yes”] Please tell me if you experienced any of the following in the past month: (Select all that apply)   - Had nightmares about these events or thought about these events when you did not want to - Tried hard not to think about the event(s) or gone out of your way to avoid situations that reminded you of the event(s) - Been constantly on guard, watchful, or easily startled - Felt numb or detached from people, activities, or your surroundings - Felt guilty or unable to stop blaming yourself or others for the event(s) or any problems the event(s) may have caused - None of the above |
| Predisposing | Homelessness History | Baseline | Team | When was the last time you had stable housing?  [Month/Year] |
| Predisposing | Criminal justice history | Baseline | Team | Have you ever spent time in jail, prison, or juvenile detention center?   a. Yes b. No |
| Predisposing | Coping strategies | Biannual | Brief Resilience Scale | People use different ways to cope with difficult times. Please think about your own experiences, such as dealing with harassment, a chronic illness, or the coronavirus pandemic. How much do the following statements describe you? [Response options: Strongly disagree; Disagree; Neither agree nor disagree; Agree; Strongly agree]   - I tend to bounce back quickly after hard times - I have a hard time making it through stressful events. - It does not take me long to recover from a stressful event. - It is hard for me to snap back when something bad happens. - I usually come through difficult times with little trouble. - I tend to take a long time to get over set-backs in my life. |
| Predisposing | Healthcare preference | Baseline & Biannual | Team | Where would you prefer to receive health care? (Select all that apply)   a. In a hospital or medical center b. In a health clinic or doctor’s office c. At an apartment/hotel/shelter d. At a mobile clinic/van e. On the street (like at your encampment or vehicle) f. Other place/location |
| Predisposing;  Secondary outcome | Health activation | Baseline & Biannual | Insignia Patient Activation Measure | Indicate how much each of the statements below describes you:  [Response options: Strongly disagree; Disagree; Agree; Strongly agree]   - Taking an active role in my own healthcare is the most important thing that affects my health - I know how to prevent problems with my health. - I can figure out solutions when new problems arise with my health. - I can maintain lifestyle changes like eating right and exercising, even when things are stressful. |
| Predisposing; Primary outcome | Trust in medical profession | Baseline & Biannual | Trust in medical profession scale (Hall 2002) | [Response options: Strongly disagree; Disagree; Neither agree nor disagree; Agree; Strongly agree]   - Medical providers in general care about their patients' health just as much or more as their patients do. - Medical providers in general will do whatever it takes to get patients all the care they need. - Sometimes medical providers care more about what is convenient for them than about their patients' medical needs. - Sometimes medical providers do not pay full attention to what patients are trying to tell them. - Medical providers always use their very best skill and effort on behalf of their patients. - A medical provider would never mislead you about anything. |
| Need;  Secondary outcome | Chronic physical disease | Baseline & Biannual | CDC list of conditions associated with COVID risk. Additional conditions added by team | Do you have any of the following medical conditions? (Select all that apply)   a. Serious heart condition b. Diabetes c. HIV/AIDS d. Cancer e. Immunocompromised f. Asthma g. COPD or other chronic respiratory disease h. Chronic kidney disease i. Blood disorder (e.g. sickle cell disease or on blood thinners) j. Liver disease (e.g. Hepatitis) k. Neurodegenerative disease (e.g. Parkinson's, MS, Huntington's) l. Hypertension m. Had a stroke in the past n. Arthritis or Osteoporosis o. Alzheimer's disease or Dementia p. Other |
| Need;  Secondary outcome | Serious mental illness | Baseline & Biannual | Team | Have you ever been told by a doctor or other health professional that you have any of the following mental health conditions? (Select all that apply)   a. Major depression b. Anxiety c. Bipolar disorder d. Schizophrenia or other psychotic disorders not related to substance use e. ADD, ADHD, or Autism f. Personality disorder such as borderline g. PTSD h. Other |
| Need;  Secondary outcome | Substance use disorder | Baseline & Biannual | Team | Have you ever been told by a doctor or other health professional that you have any of the following substance use issues? (Select all that apply)   a. Alcohol use b. Cannabis/marijuana use c. Stimulant use (including methamphetamine, cocaine) d. Opioid use (including heroin, fentanyl, prescription painkillers) e. Any/Other substance use condition, substance use disorder, or substance abuse problem |
| Need;  Secondary outcome | Disability status | Baseline & Biannual | ACS-6 | [Response options: Yes; No]   - Are you deaf, or do you have serious difficulty hearing? - Are you blind, or do you have serious difficulty seeing, even when wearing glasses? - Because of a physical, mental, or emotional condition, do you have serious difficulty concentrating, remembering, or making decisions? - Do you have serious difficulty walking or climbing stairs? - Do you have difficulty dressing or bathing? - Because of a physical, mental, or emotional condition, do you have difficulty doing errands alone such as visiting a doctor’s office or shopping? |
| Need;  Secondary outcome | Health concerns | Baseline & Biannual | Team | What has been your most important health concern over the past 6 months?   a. Mental health (e.g., depression, anxiety, bipolar disorder, PTSD, schizophrenia, etc.) b. Long-term physical health condition (e.g., cancer, high blood sugar, high blood pressure, asthma, diseases of the heart, kidney, lungs, etc.) c. Personal drug or alcohol use d. Sexually Transmitted Infections (STIs) e. Dental health f. Wounds, skin infections, or other physical injuries g. Pain h. Physical or sexual abuse i. Pregnancy-related issue j. Other issue or condition k. No health concerns   [If any health condition of concern was selected] How easy is it for you to get the medical care tests, medications, or treatment you need for your most important health concern, your [selected option]?   a. Very easy b. Easy c. Neutral d. Difficult e. Very difficult   [If any health condition of concern was selected] How would you rate the quality of the medical care you are receiving for your most important health concern, your [selected option]?   a. Poor b. Fair c. Good d. Excellent e. Not receiving any care for this condition |
| Enabling | Health insurance coverage | Baseline & Biannual | Team | Do you currently have health insurance?   a. Yes b. No |
| Enabling | Benefits | Baseline & Biannual | Team | Which of the following benefits do you currently receive? (Select all that apply)   a. General Relief (GR), Temporary Cash Assistance (TCA), Family Independence Temporary Assistance (FITAP), or Family Investment Program (FIP) b. Social Security Disability Income (SSDI) or Social Security Income (SSI) c. Retirement Income d. Supplemental Nutritional Assistance (SNAP)/CalFresh/WIC e. Temporary Assistance for Needy Families (TANF) f. Veterans’ Benefits g. CalWORKS h. Unemployment Benefits i. Other j. None of the above/Don't receive any benefits |
| Enabling;  Secondary outcome | Usual source of primary care | Baseline & Biannual | Team | Do you currently have a primary care doctor or other health provider that you usually go to when you need a check-up, are sick, or need health advice? Do not include emergency room visits.   a. Yes b. No |
| Enabling | Housing waitlist | Month 1 & Biannual | Team | Are you currently on a waiting list for housing in Los Angeles?   a. Yes b. No c. I don’t know |
| Enabling | Outreach contact | Month 1 & Biannual | Team | Since you have been in the Los Angeles area, have you had any contact with a homeless services outreach worker (i.e. from LAHSA or any other social service agency)   a. Yes b. No |
| Enabling;  Secondary outcome | Food insecurity | Monthly | USDA Food Security Module | Indicate whether any of the following happened to you in the past week (Select all that apply)   a. You didn't eat all day because there wasn't any food b. You skipped a meal because there wasn't any food c. You ate less than you should because there wasn't enough food d. You ate food that was spoiled or unsafe because there wasn't anything else to eat e. None of the above |
| Enabling;  Secondary outcome | Sanitation resources | Monthly | Housed Beds | In the past week, how easy or hard was it for you to:  [Response options: Very easy; Easy; Neither easy nor hard; Hard; Very hard]   - Find a toilet when you needed to use the bathroom - Find a place to shower or wash up - Find clean water for drinking or cooking |
| Enabling | Employment | Monthly | Team | In the past 7 days, did you do any work?   a. Yes b. No |
| Enabling | Location | Monthly | Team | [Participants can choose to provide their exact coordinate location, cross streets, or neighborhood they slept at last night] |
| Enabling | Residential stability | Monthly | Team | In the past 30 days, how many times did you move locations where you slept (i.e., moving from one address or street to another)? |
| Secondary outcome | Health care utilization | Monthly | Team | Did you receive any health or medical care in the past 30 days?  This can include any services you received for your physical health, sexual health, pregnancy, drug and alcohol use, and mental health.   a. Yes b. No |
| Field medicine engagement | Field medicine utilization | Monthly | Team | [If selected “Yes” for receiving health or medical care in the past 30 days] Where did you receive this care (Select all that apply) Emergency room   a. Urgent care center or drop-in clinic (i.e., no appointment was needed) b. A scheduled appointment at a doctor’s office, clinic, medical center, or hospital c. At an apartment, hotel, or shelter d. At a mobile clinic / van that you went to e. On the street (like at your encampment or vehicle) f. Other |
| Field medicine engagement | Field medicine services and satisfaction | Monthly | Team | [If selected “At an apartment, hotel, or shelter”, “At a mobile clinic/van that you sent to”, or “On the street” for where they received care]  What health or medical care did you receive? (Select all that apply)   a. Check-up of your vital signs (i.e., body temperature, blood pressure, pulse/heart rate, and breathing). b. Wound care c. Giving or helping you get your medication d. Treatment for long-term medical condition(s) you have (e.g., high blood sugar, high blood pressure, cancer, heart disease) e. Help to get other medical services or treatment f. Treatment for substance use or safer drug use (e.g., medication, clean needles, Narcan) g. Treatment for mental health condition(s) you have h. Getting you tested for a health condition (e.g., STIs, heart disease) i. Other   Was there any health or medical care that you wanted or needed but did NOT receive during these visits?   a. Yes b. No   What other services, supplies, or help did you receive during these visits? (Select all that apply)   a. Social services (like applying for ID or for government benefits) b. Housing services (like applying for Section 8 or shelter) c. Food and/or water d. Hygiene kits e. Clothing f. Money, gift cards, vouchers g. Transit passes h. Cell phones i. Other j. None of the above   How would you rate your satisfaction with any of the health care or social services you received at a mobile clinic/van, encampment, shelter, or on the street?   a. Poor b. Fair c. Good d. Very good e. Excellent   Please select all the statements that you AGREE with about the health care team you saw during your visits [at a mobile clinic or van/ on the street]:   a. They always or constantly show up when they say they will b. I can reach them if I need something related to my health c. They listen to me and respect my decisions d. They have connected me with or provided me with the care I need to be healthier e. They are warm and empathetic f. They spend an appropriate amount of time with me g. They  are knowledgeable about taking care of my health problems h. They  work well as a team i. I trust them j. None of the above |
| Secondary outcome | Ability to contact field medicine provider | Monthly | Team | [If selected “At an apartment, hotel, or shelter”, “At a mobile clinic/van that you sent to”, or “On the street” for where they received care]  Would you know how to contact your this [field medicine] provider if you needed medical attention?   a. Yes, I have a way to call, text, or email them b. Yes, I could walk into their facility c. Yes, I know when they are out on the street or in shelters d. Not sure e. No |
| Primary outcome | ER visits | Monthly | Team | How many times have you been to the emergency room in the past month? |
| Primary outcome | Hospital visits | Monthly | Team | How many nights have you spent in a hospital in the past month? |
| Primary outcome | Physical health status (current) | Monthly | Patient Reported Outcomes Measurement Information System (PROMIS) | In general, how would you rate your physical health?   a. Poor b. Fair c. Good d. Very good e. Excellent |
| Primary outcome | Bodily pain | Monthly | Medical Outcomes Study (MOS) Physical Functioning Measure | How much bodily pain have you had during the past 4 weeks?   a. None b. Very mild c. Mild d. Moderate e. Severe f. Very severe |
| Primary outcome | Psychological distress | Monthly | Patient Health Questionnaire (PHQ-4) | Over the last two weeks, how often have you been bothered by the following problems?  [Response options: Not at all; Several days; More than half the days; Nearly every day]   - Feeling nervous, anxious, or on edge - Not being able to stop or control worrying - Feeling down, depressed or hopeless - Little interest or pleasure in doing things |
| Primary outcome | System navigation support | Monthly | Team | In the past 30 days, did you receive any help from an outreach worker, social worker, or health care professional in getting or applying for: (Select all that apply)   a. Government issued ID or driver’s license b. Health insurance c. General Relief or other cash assistance program d. Food stamps (e.g., Supplemental Nutritional Assistance (SNAP)/CalFresh/WIC) e. Social Security Disability Income (SSDI) or Social Security Income (SSI) f. Shelter, emergency/temporary housing, or a motel/hotel provided by an agency g. Section 8 Housing program h. Other i. None |
| Primary outcome | Housing offers (Past month) | Monthly | Team | In the past 30 days, have you been offered a housing or shelter option by an outreach worker or other government/city worker?   a. Yes b. No |
| Primary outcome | Outreach contact (Past month) | Monthly | Team | In the past 30 days, have you had any contact with a homeless services outreach worker (i.e. from LAHSA or any other social service agency)   a. Yes b. No |
| Primary outcome | Last night housing status | Monthly | Team | Where did you sleep last night?   a. In shelter, emergency/temporary housing/tiny home, or hotel provided by an agency b. Outside c. Tent or makeshift shelter d. In a bus station, train station, airport e. Abandoned building f. In a vehicle (car, van, truck, RV) g. My own apartment or home h. Someone else's apartment or home i. Hotel that I paid for j. In an institution, hospital or facility |
| Primary outcome | Last month housing status | Monthly | Team | Please indicate all places where you have slept for at least one night in the past 30 days (Select all that apply)   a. In shelter, emergency/temporary housing/tiny home, or hotel provided by an agency b. Outside c. Tent or makeshift shelter d. In a bus station, train station, airport e. Abandoned building f. In a vehicle (car, van, truck, RV) g. My own apartment or home h. Someone else's apartment or home i. Hotel that I paid for   In an institution, hospital or facility |
| Secondary outcome | Unmet health care need | Monthly | Team | Have you ever wanted or needed health care in the past 30 days but did not get it?   a. Yes b. No |
| Secondary outcome | Missed medications | Monthly | Team | Have you missed taking any prescribed medications in the last 7 days?   a. Yes b. No |
| Secondary outcome | Smoking | Monthly | Team | Do you currently smoke cigarettes?   a. Yes, regularly b. Yes, occasionally c. Not at all |
| Secondary outcome | Alcohol use | Monthly | Alcohol Use Disorders Identification Test (AUDIT-C) | [Response options: Never; Once or twice in a month; Once a week; 2 to 3 times a week; 4 to 6 times a week; Daily]  In the last month, how often did you have a drink containing alcohol?  [If “Never” is not selected] In the last month, how often did you have 4 or more drinks on one occasion? |
| Secondary outcome | Marijuana use | Monthly | WHO The Alcohol, Smoking and Substance Involvement Screening Test (ASSIST) | [Response options: Never; Once or twice in a month; Once a week; 2 to 3 times a week; 4 to 6 times a week; Daily]  In the last month how often did you take cannabis/marijuana?  [If “Never” is not selected] In the last month how often were you so high from taking cannabis/marijuana that you were a danger to yourself or others? |
| Secondary outcome | Illicit drug use | Monthly | WHO The Alcohol, Smoking and Substance Involvement Screening Test (ASSIST) | [Response options: Never; Once or twice in a month; Once a week; 2 to 3 times a week; 4 to 6 times a week; Daily]  In the last month how often did you take substances (other than cannabis/marijuana), like meth, cocaine, fentanyl, heroin, prescription opioids, etc?  [If “Never” is not selected] In the last month how often were you so high from taking these substances (i.e., meth, cocaine, fentanyl, heroin, prescription opioids; NOT cannabis/marijuana) that you were a danger to yourself or others? |
| Secondary outcome | Sleep quality | Monthly | PROMIS Sleep Disturbance | How many hours of sleep did you get in the last 24 hours, including naps?  How would you rate your quality of sleep in the past week?   a. Very poor b. Poor c. Fair d. Good e. Very good |

Note: Prefer not to answer was a response option for all questions
