# Supplementary material for: Systemwide Evaluation of Field Medicine Programs in Los Angeles County: Protocol for a Mixed Methods Longitudinal Observational Study
Source: JMIR Res Protoc. 2026 Oct 14;15:e94248. doi: 10.2196/94248 (PMC13664992; doi:10.2196/94248)
Supplement: Supplementary file 2 [file resprot-v15-e94248-s002.doc]

**Completed STROBE checklist for cohort studies**

This checklist was elaborated using formal items recommended for cohort studies from STROBE statement ([https://www.strobe-statement.org](https://www.strobe-statement.org/)).

|  | Item No | Recommendation | Our paper |
| --- | --- | --- | --- |
| **Title and abstract** | 1 | (*a*) Indicate the study’s design with a commonly used term in the title or the abstract | Study design is indicated in the title and abstract |
|  |  | (*b*) Provide in the abstract an informative and balanced summary of what was done and what was found | N/A (this paper is a protocol paper) |
| Introduction | | |  |
| Background/rationale | 2 | Explain the scientific background and rationale for the investigation being reported | Rationale and existing literature are stated in the introduction  section |
| Objectives | 3 | State specific objectives, including any prespecified hypotheses | A statement is provided at the end of the introduction (subsection “Theoretical Framework, Research Questions, and Hypotheses”) that specifies the objectives and hypotheses |
| Methods | | |  |
| Study design | 4 | Present key elements of study design early in the paper | Study design is stated in the first sentence in the Methods section. Key elements are all described in the methods.  “This evaluation uses a mixed-methods design, drawing on quantitative survey data from a comparative longitudinal cohort study and qualitative data from field observations and semi-structured interviews.” |
| Setting | 5 | Describe the setting, locations, and relevant dates, including periods of recruitment, exposure, follow-up, and data collection | Setting, contexts, dates of inclusion, are fully described in the method section |
| Participants | 6 | (*a*) Give the eligibility criteria, and the sources and methods of selection of participants. Describe methods of follow-up | Eligibility criteria and sources and methods of selection of participants are provided in the “Recruitment of study participants” subsections for both the Quantitative and Qualitative Research Methods.  Methods of follow-up is only applicable for the Quantitative Research Method, and is described in the following sentence: “On completion of the baseline survey, they are sent a survey link each month via text or email” |
|  |  | (*b*) For matched studies, give matching criteria and number of exposed and unexposed | Matching criteria is described in the subsection “Statistical Adjustments for Non-random Assignment”. Numbers are not yet known since this is a protocol paper |
| Variables | 7 | Clearly define all outcomes, exposures, predictors, potential confounders, and effect modifiers. Give diagnostic criteria, if applicable | Details provided in the “Quantitative Measures” subsection. |
| Data sources/ measurement | 8* | For each variable of interest, give sources of data and details of methods of assessment (measurement). Describe comparability of assessment methods if there is more than one group | Data collection and measurement was the same for all variables,  and is described in the “Quantitative Measures” subsection. The exact items and sources for these variables are provided in Multimedia Appendix 1. |
| Bias | 9 | Describe any efforts to address potential sources of bias | The “Statistical Adjustments for Non-random Assignment” subsection describes efforts address potential sources of bias from non-random assignment and selective attrition. |
| Study size | 10 | Explain how the study size was arrived at | The method describes the recruitment of participants through different programs and modes over several years. |
| Quantitative variables | 11 | Explain how quantitative variables were handled in the analyses. If applicable, describe which groupings were chosen and why | The “Quantitative Analysis Methods” subsection describes how variable are planned to be handled in the analysis.  Definitions of all categories for variables are presented in Multimedia Appendix 1. |
| Statistical methods | 12 | (*a*) Describe all statistical methods, including those used to control for confounding | These are described in the method section for each aim. |
|  |  | (*b*) Describe any methods used to examine subgroups and interactions | N/A |
|  |  | (*c*) Explain how missing data were addressed | Described in the “Quantitative Analysis Methods” section: “As the PATHS survey includes a “prefer not to answer” option for all questions, we anticipate a relatively high level of missingness and, thus, will conduct all analyses using first a complete case analysis followed by a complete sample using multiple imputation methods. We presume that analyses will primarily use imputed data, while complete case analysis will serve as a robustness check.” |
|  |  | (*d*) If applicable, explain how loss to follow-up was addressed | The last paragraph of “Statistical Adjustments for Non-random Assignment” subsection describes how we plan to address loss to follow-up and selective attrition. |
|  |  | (*e*) Describe any sensitivity analyses | Different sensitivity analyses are described throughout the “Quantitative Analysis Methods” subsection. |
| Results | | |  |
| Participants | 13* | (a) Report numbers of individuals at each stage of study—eg numbers potentially eligible, examined for eligibility, confirmed eligible, included in the study, completing follow-up, and analysed | As this is a protocol paper, we provide the numbers potentially eligible, examined for eligibility, confirmed eligible, included in the study. We do not yet information of those completing follow-up and who will be analysed. |
|  |  | (b) Give reasons for non-participation at each stage | N/A |
|  |  | (c) Consider use of a flow diagram | N/A |
| Descriptive data | 14* | (a) Give characteristics of study participants (eg demographic, clinical, social) and information on exposures and potential confounders | N/A (not available for this protocol paper) |
|  |  | (b) Indicate number of participants with missing data for each variable of interest | N/A (not available for this protocol paper) |
|  |  | (c) Summarise follow-up time (eg, average and total amount) | N/A (not available for this protocol paper) |
| Outcome data | 15* | Report numbers of outcome events or summary measures over time | N/A (not available for this protocol paper) |
| Main results | 16 | (*a*) Give unadjusted estimates and, if applicable, confounder-adjusted estimates and their precision (eg, 95% confidence interval). Make clear which confounders were adjusted for and why they were included | N/A |
|  |  | (*b*) Report category boundaries when continuous variables were categorized | N/A |
|  |  | (*c*) If relevant, consider translating estimates of relative risk into absolute risk for a meaningful time period | N/A |
| Other analyses | 17 | Report other analyses done—eg analyses of subgroups and interactions, and sensitivity analyses | N/A |
| Discussion | | |  |
| Key results | 18 | Summarise key results with reference to study objectives | Not available for this protocol paper. Instead we provide description of “Anticipated Findings” in the Discussion section |
| Limitations | 19 | Discuss limitations of the study, taking into account sources of potential bias or imprecision. Discuss both direction and magnitude of any potential bias | Description of limitations is provided in the “Limitations” subsection of the Discussion. |
| Interpretation | 20 | Give a cautious overall interpretation of results considering objectives, limitations, multiplicity of analyses, results from similar studies, and other relevant evidence | N/A |
| Generalisability | 21 | Discuss the generalisability (external validity) of the study results | N/A |
| Other information | | |  |
| Funding | 22 | Give the source of funding and the role of the funders for the present study and, if applicable, for the original study on which the present article is based | A funding statement is provided in the manuscript. |

*Give information separately for exposed and unexposed groups.
